# An update on screen time and choice reaction time among medical college students in India

**DOI:** 10.6026/973206300212096

**Published:** 2025-07-31

**Authors:** Meraj Ahmad, Jinsha J.S, Amrit Pal Singh Virk, Mogis Ahmad, Sachin Parmar

**Affiliations:** 1 Department of Physiology, Government Medical College, Datia, Madhya Pradesh, India; 2 Department of Physiology, MGM Medical College, Indore, Madhya Pradesh, India; 3 Department of Community Medcine V.K.S. Government Medical College, Neemuch, Madhya Pradesh, India

**Keywords:** Screen time, cognitive performance, choice reaction time (CRT), sleep duration, college students

## Abstract

There is an increased screen time among medical college students in India. This is a huge concern. Therefore, it is of interest to report an update on the relationship between screen time and cognitive performance, specifically choice reaction time (CRT), among 147 undergraduate students aged 18-25 years. The results indicate a significant positive correlation between increased screen time and prolonged CRT, suggesting that excessive screen use negatively affects cognitive responsiveness. Additionally, screen time was found to be inversely correlated with sleep duration, pointing to disrupted sleep as a potential mediator for cognitive decline. No significant gender differences were observed in CRT, highlighting the universal impact of screen time on cognitive function. These findings emphasize the need for balanced screen use and adequate sleep to maintain optimal cognitive performance in young adults.

## Background:

The digital revolution has fundamentally transformed the daily lives of college students, with digital devices becoming ubiquitous tools for academic, social, and recreational activities. University students today spend an unprecedented amount of time engaged with electronic screens, with recent studies reporting average daily screen exposure ranging from 7.1 to 14.3 hours per day [1, 2] This dramatic increase in screen time has raised significant concerns among researchers and educators regarding its potential impact on cognitive functioning, particularly in areas critical for academic success and daily performance. Screen time, defined as the duration spent viewing electronic displays including smartphones, tablets, computers, and televisions, has become a dominant aspect of modern life [3]. Among college populations, this exposure is particularly intensive, with students utilizing digital devices for studying, communication, entertainment, and information processing. Research indicates that university students with smartphone addiction exceed 8 hours of daily screen time, while even those without addiction maintain similarly high usage patterns [4]. The pervasive nature of digital media exposure has created an unprecedented experimental condition for understanding its effects on human cognitive processes. Choice reaction time represents a fundamental measure of cognitive processing speed and executive function, reflecting the duration required for an individual to respond to multiple stimuli while discriminating between them and selecting appropriate responses [5]. Unlike simple reaction time, which involves responding to a single stimulus with a predetermined response, choice reaction time tasks require participants to process multiple stimuli, make decisions, and execute specific responses based on stimulus characteristics [6]. This cognitive measure is particularly relevant for college students, as it reflects the type of rapid decision-making and information processing required in academic and professional environments.

The theoretical framework underlying choice reaction time encompasses several cognitive processes, including stimulus detection, perceptual discrimination, response selection, and motor execution [7]. Mental chronometry research has established that choice reaction time tasks typically yield response times between 350-450 milliseconds for healthy young adults, significantly slower than simple reaction times of approximately 160-190 milliseconds [8]. This difference reflects the additional cognitive load imposed by decision-making processes, making choice reaction time a sensitive indicator of executive function and processing efficiency. Emerging research suggests concerning associations between excessive screen time and various aspects of cognitive functioning. Studies utilizing neuroimaging techniques have demonstrated that screen time exposure causes structural and functional changes in brain regions critical for executive functions, including the prefrontal cortex, which underlies working memory, planning, and cognitive flexibility [9, 10]. These neurological alterations may have direct implications for cognitive performance measures such as choice reaction time, as the affected brain regions are integral to the rapid decision-making processes required in choice reaction paradigms. Recent investigations among university populations have revealed significant negative correlations between screen time and cognitive performance measures. A comprehensive study of 305 medical students found that excessive screen time was associated with reduced fluid intelligence, impaired attention, and slower cognitive processing, with neurophysiological measures indicating prolonged latencies in event-related potentials critical for attention and working memory [11]. These findings suggest that the cognitive resources essential for rapid decision-making may be compromised in individuals with high screen exposure.

The mechanisms underlying the relationship between screen time and cognitive performance appear multifaceted. Excessive digital media exposure has been linked to attention deficits, reduced concentration spans, and impaired executive functioning [12, 13]. Studies have documented that prolonged screen exposure can lead to cognitive inflexibility, increased impulsivity, and decreased decision-making ability [14]. These cognitive changes may directly impact choice reaction time performance by interfering with the rapid stimulus processing, response selection, and executive control processes required for optimal performance. Furthermore, screen time exposure has been associated with disruptions in sleep patterns, which may indirectly affect cognitive performance [15]. The blue light emission from digital devices can interfere with circadian rhythms and melatonin production, leading to reduced sleep quality and duration. Given that adequate sleep is essential for optimal cognitive functioning and reaction time performance, sleep disruption represents an important mediating factor in the screen time-cognition relationship. The college student population represents a particularly vulnerable group for screen time effects, as they are in a critical period of neural development and face high cognitive demands. University students must simultaneously process large amounts of information, make rapid decisions, and maintain sustained attention across multiple academic and social contexts [16]. The potential impairment of choice reaction time due to excessive screen exposure could therefore have significant implications for academic performance, learning efficiency, and overall cognitive development. Current research gaps exist in understanding the specific relationship between screen time and choice reaction time in adult college populations. While studies have examined general cognitive effects of screen exposure and established normative values for choice reaction time, few investigations have directly examined this association in university settings. Additionally, the optimal thresholds for screen time exposure that may preserve cognitive function remain poorly defined; with some research suggesting that exposure exceeding 6.5 hours daily may be particularly detrimental [3]. An initial analysis of screen time and choice reaction time is available [25]. Therefore, it is of interest to report the association between screen time exposure and choice reaction time performance in adult college students.

## Materials and Methods:

## Study design and setting:

This cross-sectional observational study was conducted at Mahatma Gandhi Memorial Medical College, Indore and Madhya Pradesh. The study aimed to investigate the relationship between screen time exposure and cognitive performance among undergraduate students.

## Participants:

A total of 147 undergraduate students, aged between 18 and 25 years, were selected through a convenience sampling method. Participants were required to meet the inclusion criteria to be eligible for participation.

## Inclusion criteria:

[1] Regular users of screens, including smartphones, laptops, and tablets.

[2] Willingness to provide informed consent for participation in the study.

[3] Age between 18 and 25 years.

## Exclusion criteria:

[1] Individuals with known neurological or psychiatric disorders.

[2] Participants using medications that may affect cognitive function, such as sedatives or stimulants.

## Data collection procedures:

[1] Screen time assessment: Participants were asked to self-report their average daily screen exposure in hours per day for the past week. The data was collected via a questionnaire designed to capture the frequency and duration of screen use across various devices such as smartphones, laptops, and tablets.

[2] Cognitive performance measurement (CRT): Cognitive performance was assessed using the Deary-Liewald Reaction Time Task, a validated computer-based tool that measures reaction times as an indicator of cognitive processing speed. The task was administered in a quiet environment to minimize distractions and ensure accurate results.

## Ethical considerations:

The study was approved by the institutional ethics committee, with clearance obtained under the number EC/MGM/Dec-22/32. All participants provided written informed consent prior to data collection, ensuring their voluntary participation and confidentiality of their responses.

## Results:

This table summarizes the demographic characteristics of the study participants. The mean age was 20.0 ± 2.25 years. The sample consisted of 76 males (51.7%) and 71 females (48.3%). The average height was 166.37 ± 8.33 cm, mean weight was 58.79 ± 9.97 kg, and the mean BMI was 21.21 ± 3.09, indicating that most subjects were within the normal weight range (Table 1 - see PDF). This table presents various physiological and lifestyle parameters. The mean systolic blood pressure (SBP) was 117.69 ± 5.87 mmHg, and diastolic blood pressure (DBP) was 77.44 ± 4.93 mmHg, resulting in a pulse pressure (PP) of 40.24 ± 4.73 mmHg. The mean pulse rate (PR) was 77.14 ± 5.00 bpm. Sensory reaction time (SRT) and choice reaction time (CRT) were 285.56 ± 68.67 ms and 500.16 ± 120.72 ms respectively. The average screen time (ST) was 2.75 ± 0.78 hours, while mean sleep duration was 6.25 ± 1.16 hours (Table 2 - see PDF). This table shows a statistically significant positive correlation between screen time and both SRT (rho = 0.436, p < 0.0001) and CRT (rho = 0.621, p < 0.0001), indicating longer screen time is associated with slower reaction times. Conversely, screen time had a strong negative correlation with sleep duration (rho = -0.738, p < 0.0001), suggesting that increased screen time is linked to reduced sleep (Table 3 - see PDF). This table compares males and females across various physiological and lifestyle variables. Males were significantly taller and heavier than females (p < 0.0001 for both), though BMI differences were not statistically significant (p = 0.296). Males had higher SBP and DBP (p = 0.002 and <0.0001, respectively), while there were no significant gender differences in PP, PR, SRT, CRT, or screen time. However, females reported significantly longer sleep duration compared to males (p = 0.044) (Table 4 - see PDF).

## Discussion:

The current study reveals a significant positive correlation between screen time and choice reaction time (CRT) among college students (rho = 0.621, p < 0.0001), suggesting that increased screen exposure is associated with slower reaction times. These findings align with several existing studies that have explored the relationship between digital screen use and cognitive performance. A comprehensive study involving 145 healthy young adults (mean age 21.55 ± 2.84 years) specifically examined the impact of night screen time on cognitive function [17]. The researchers found that increased night screen time was associated with lower scores on cognitive tests measuring information processing speed, working memory, calculation, and attention domains. This study particularly emphasized that night screen exposure had more detrimental effects on cognitive performance compared to daytime screen use, which may explain the stronger correlation observed in the current study. The negative correlation between screen time and sleep duration (rho = -0.738, p < 0.0001) found in the current study is strongly supported by recent research from Norway involving 45,202 university students aged 18-28 years [18]. This large-scale study demonstrated that each additional hour of screen time after bedtime increased the odds of insomnia by 59% and reduced sleep duration by 24 minutes. The researchers concluded that screen use displaces sleep by taking up time that would otherwise be spent resting, which directly impacts cognitive performance including reaction time. Several studies have explored the mechanisms behind these effects, with research indicating that prolonged screen exposure may impair cognitive functions through multiple pathways. A systematic review examining screen time's impact on attention abilities found that high levels of screen exposure are harmful to attentional functions, particularly in young populations [19]. This finding supports the current study's observation that increased screen time correlates with slower reaction times, as both attention and reaction time are closely linked cognitive functions.

The absence of significant gender differences in CRT in the current study (503.20 ± 125.46 ms vs. 496.90 ± 116.25 ms for males vs. females, p = 0.753) is consistent with some previous research, though the literature shows mixed results regarding gender differences in reaction time. A study examining visual reaction time in medical students found that males had significantly faster reaction times than females for both simple and choice visual reactions [20]. However, the current study's findings suggest that the impact of screen time on cognitive performance may be more uniform across genders in the college-age population. The theoretical framework for understanding these effects is supported by research on the "brain drain" phenomenon, where the mere presence of smartphones can reduce available cognitive capacity [21]. Studies have shown that smartphone presence impairs cognitive performance by occupying limited attentional resources, even when the devices are not actively being used. This mechanism may explain why students with higher screen time exposure demonstrate slower reaction times, as their cognitive resources are continuously allocated to managing digital distractions. Furthermore, research on media multitasking has revealed that heavy media multi-taskers show reduced cognitive control and are more susceptible to interference from irrelevant stimuli [22]. A large-scale study involving participants aged 7-70 years found that higher levels of everyday technology multitasking were associated with changes in cognitive flexibility across different age groups [23]. This finding is particularly relevant to the current study's population of college students, who are likely to engage in frequent media multitasking behaviors. The current study's emphasis on the need for screen time moderation is reinforced by research showing that excessive digital device usage during academic settings can negatively impact learning outcomes [24]. Studies have demonstrated that students who used only one application during lecture time achieved higher academic performance compared to those who multitasked with multiple applications, suggesting that digital distractions can significantly impair cognitive processing and academic achievement.

## Conclusion:

The research identifies a relevant dependence between heightened screen-time versus a lengthened choice reaction time (CRT) indicating an adverse influence on cognitive responsiveness. It also discovers a positive relationship between screen time and sleep deprivation, meaning disturbed sleeping behavior might be one of the causes of cognitive decline. These results point to the importance of moderate use of screens and adequate sleep patterns in the promotion of cognitive well-being among young people.
